# A multidimensional quality model: an opportunity for patients, their kin, healthcare providers and professionals in the new COVID-19 period

**DOI:** 10.12688/f1000research.26368.1

**Published:** 2020-09-16

**Authors:** Peter Lachman, Paul Batalden, Kris Vanhaecht

**Affiliations:** 1 International Society for Quality in Health Care (ISQua), Dublin, D02 NY63, Ireland; 2 Dartmouth Institute for Health Policy and Clinical Practice, Dartmouth College, Lebanon, NH, USA; 3 KU Leuven Institute for Healthcare Policy, KU Leuven, Leuven, 3000, Belgium; 4 Department of Quality, University Hospitals Leuven, Leuven, 3000, Belgium

**Keywords:** quality, safety, kin centered, covid19, person centered care

## Abstract

**Background:** It is twenty years since the Institute of Medicine (IOM) defined quality in healthcare, as comprising six domains: person-centredness, timeliness, efficiency, effectiveness, safety and equity. Since then, a new quality movement has emerged, with the development of numerous interventions aimed at improving quality, with a focus on accessibility, safety and effectiveness of care. Further gains in equity and timeliness have proven even more challenging.

**The challenge:** With the emergence of “service-oriented” systems, complexity science, the challenges of climate change, the growth of social media and the internet and the new reality of COVID-19, the original domains proposed by the IOM invite reflection on their relevance and possibility for improvement.

**The possible solution: **In this paper we propose a revised model of quality that is built on never-ending learning and includes new domains, such as Ecology and Transparency, which reflect the changing worldview of healthcare. We also introduce the concept of person- or “kin-centred care” to emphasise the shared humanity of people involved in the interdependent work. This is a more expansive view of what “person-centredness” began. The delivery of health and healthcare requires people working in differing roles, with explicit attention to the lived realities of the people in the roles of professional and patient. The new model will provide a construct that may make the attainment of equity in healthcare more possible with a focus on kindness for all.

## The rationale for change

Over the past twenty years, since the defining of quality in healthcare by the Institute of Medicine (IOM) ^1^, an industry has developed in the field of quality improvement and patient safety. This has included the academic study of the theory and methodology and the actual implementation of the studied theory. The result has been some improvement, but not to the extent that would allow a claim of success ^2, 3^. It has been said that there is insufficient evidence for the impact of quality improvement and more research is required ^4^. In this paper, we take the opportunity to revise our basic framework and to redefine quality with the advantage of the experience gained over the past 20 years. One may ask why we need to redefine what is meant by quality in healthcare.

The actual work of healthcare service today struggles to meet the needs of people for better health. It has been designed to address failures in disease management, rather than in working with people to maintain health. It seems easier to focus on “standard work” and the “actions” in disease management, rather than on a more integrated view of the “relationships” that are required to maintain health. Furthermore, more advances in health have come from preventive measures in public health, such as immunisation, clean water, sanitation and housing ^5, 6^. In addition, the methods of assessing the impact of quality improvement have not lent themselves well to the standard way of assessing interventions in healthcare, nor have they bridged the gap between disease management and wellness or disease prevention ^7^.

Current healthcare service improvement has adopted many theories, methodologies and interventions from other industries, which have demonstrated important gains in quality, cost and safety. During the last century, one can discern two approaches on the creation, assessment, and improvement of the quality of healthcare delivery (see Table 1). Each approach has made important contributions to our abilities to make a better healthcare service and each has worked around a relatively common question. For convenience, we have named the first approach, Quality 1.0, “Q 1.0”. This began in the second decade of the 20th century in the USA, when the American College of Surgeons began their program of hospital standards. Three decades later, other national organizations of hospitals and professionals joined to form the “Joint Commission” for the Accreditation of Hospitals ^8, 9^. With the passage of the Medicare payment program, these certification efforts were linked to qualification for receipt of payment for hospitalisation.

With the advent of post-World War II improvement in systems thinking and system improvement methods, system- or enterprise-wide efforts to address quality emerged in many economic sectors. Initially, these improvement initiatives occurred outside of healthcare service, but increasingly from the mid-1980’s, improvement interventions spread to healthcare services. This new approach is termed Quality 2.0, “Q 2.0”. In this process the ideas of quality were defined by Donabedian as being system- and process-driven to produce the desired outcomes ^10^. The early interventions to make quality a system or enterprise-wide endeavour were promoted with the introduction of the theories and methods of W. Edwards Deming, Joseph M. Juran and others ^11– 14^.

**Table 1.** Stages of quality improvement in healthcare.

Quality 1.0	Quality 2.0	Quality 3.0
*Thresholds*	*Organization-wide systems*	*Coproduction of health*
“How might we establish thresholds for good healthcare service?”	“How might we use ‘enterprise- wide systems’ for best disease management?”	“How might we improve the value of the contribution that healthcare service makes to health?”
Illustrative themes: ● Development of Standards ● Inspection to assess ● Certification ● Guidelines	Illustrative themes: ● Systems, processes ● Reliability ● Customer-supplier ● Performance measurement	Illustrative themes: ● Logic of making a “service” ● Ownership of “health” ● Kinship of coproducing people ● Integration of multiple knowledge systems ● Value-creating system architecture

The IOM provided an important stimulus for the current focus on quality in healthcare with its reviews of the safety and quality of health care services ^1, 15^. The IOM defined six domains of quality, which have become the standard within the growing development of the science of improvement in healthcare: safe, efficient, effective, timely, equitable and patient-centred ^1^. The theories and methodologies that had been successful in other economic sectors have been thought to be appropriate to the challenges of quality in health care delivery ^16– 18^. We have learned much, as a new language of systems, processes and outcomes has been added to the study and practice of clinical excellence, previously thought to be “quality in healthcare.” Attention shifted from a minimum “threshold” of quality to the concept of a “ceiling” of quality—not, “ *are you good enough to qualify?*” but “ *how good can quality become?*” Examples of success have been decreases in some infection rates, perceived increased access to healthcare, changes in person-centred care and improvements in aspects of safety ^19– 23^. System-wide improvement has been demonstrated at some institutions ^24^.

Yet, for all these achievements, the persistence and the universal nature of the problem was highlighted in three key publications in 2018, which demonstrated that more than eight million people die from poor quality care in low and middle income countries ^25– 27^. In high income countries, at least 1 out of 10 patients is adversely affected during treatment, often resulting from persistent unwarranted variations in healthcare delivery, where a considerable proportion of patients did not receive appropriate, evidence-based care ^28^.

We believe that the development of technical solutions helped connect improvement efforts to the earlier focus on “professional work.” These efforts allowed many gains. For example, specific safety initiatives have decreased pressure ulcers, falls in hospitals and hospital-acquired infections ^29– 32^. However, today we can also recognise the diminishment of attention to some very basic issues. For example, what does “quality” really mean to the person whose health it is? In our efforts to clarify desired professional roles, we may have inadvertently created a “product-dominant logic”: professionals making a quality healthcare service and then trying to “sell” it to patients. We think it is time to step back and reconsider what healthcare service is. How is it made and what does quality really means to the person whose health it is?

The approach has changed more recently, and the focusing question seems to have become something of the following nature: “How might we improve the value of the contribution that healthcare service makes to health?” ^33^. This invites attention to who actually owns a person’s health: the healthcare provider or the individual receiving healthcare? In addition, we postulate that the concept of kinship extends to include both the care giver and the care provider, as they work together to make and improve services in support of an individual’s or a community’s health. The work of design, execution, assessment and improvement involves the integration of multiple systems of knowledge and skill.

Co-productive work invites new models of value creation and attention to the basic architecture of those systems. Because these are different to those in the approach of “Q 2.0,” we have named this approach Quality 3.0, “Q 3.0”. Each of these approaches to quality offer important insights into the complex work involved in healthcare service. We think of each approach as adding to our capability to make better health, rather than “substituting” or “replacing” for the earlier approaches. The approaches are summarised in Table 1.

In this paper, we propose a new construct for defining quality of healthcare, where the aim is to meet the needs of the patient as a person, rather than meeting the needs of the healthcare system, which is as complex industry selling a product of disease management ^34^. In proposing a new framework, it is tempting to dismiss earlier concepts. While we utilise the same dimensions, they have been reoriented with new ones added to invite a “service-dominant” logic. The new dimensions of quality will become even more relevant for the way we will facilitate health and make healthcare services in the future.

## Why now?

Many forces are at work today that seem to invite these changes. Information access has become more open, with the growth of the internet and social media, so it is much easier for any person to explore what is known about a problem or condition. “Making” and the maker-society invite a sense of personal agency more than traditional deference to “professional experts.” Healthcare professionals have been working to shed paternalistic legacies, creating a new construct, which we have named the commons, whereby all are working together towards the common good of health rather than simply managing disease. This is evidenced in some of the interventions to address the challenge of COVID-19. Historic conventions about payment and finance have given way to significant organizational financial stresses in all societies. The challenge of explicitly recognising the contributions of patients and families, in addition to those of professionals, while maintaining a person-centred focus during and after the pandemic for people who are affected and for those who are not, has invited a new model of quality for the future.

Concurrent with the pandemic, the issue of the structural inequalities in society have become more prominent. A new model is required to address the way we, as healthcare providers, address issues in society that impact the health of the people. These include structural racism ^35^ and the social determinants of health ^36^, including food insecurity ^37^, gender inequality ^38^ and inherent violence ^39, 40^ within many societies. COVID-19 has unmasked these, and we think the new model is a response to the past failures of society to address these issues. Some may say that this is politicisation of health. Rather we see it as making the quality model socially relevant to our times and to the people who are most marginalised.

One of the early developers of modern Health Services Research, Kerr White, noted that the public’s health was not well served by the schism that developed during the last century between “medicine” (personal health) and “public health” ^41^. He suggested that this separation was not serving the public’s health well and that the study of epidemiology might help. Today, the challenge of the COVID-19 pandemic has given us another clear view of the ways that this separation has had real consequences in unnecessary death and continues to serve us poorly. We believe that an appreciation of the common humanity—kin—amongst the people who act in the personal and in the public sectors, in addition to the study and contribution of epidemiology, can help. This focus on the relationships helps energise a bridge across the divide of the two sectors. By an explicit focus on the concept of kin, we can see a person as an individual and as a member of a population. This shared position of people helps us appreciate that kin-shipness or “kindness” can serve as a core value. It has helped us recognise the importance of kin, our fellow human beings, in our daily lives and that the absence of attention to these relationships—kin—, is a painful limitation to how we pursue health, not only in COVID-19, but also in numerous other ways, including in the end of life, for example. By kin we refer to the wider social construct around the people involved in receiving and providing care. Moreover, there is a need to develop a new way of thinking as one faces the challenges of measuring wellness, equity and good health ^42^. The COVID-19 pandemic has exposed the failure of linear thinking to produce results when responding to a crisis. This has demonstrated that we need to see quality as part of a complex adaptive system with many competing linkages. Healthcare has many components, both within the formal structures of health service delivery and more importantly within the community and in other sectors. To produce health, these components need to interact in a way that benefits the people receiving care ^43, 44^.

In short, we can now see clearly that not only is it very difficult to outsource one’s health to someone else—the truth is that we have no real option but to work in new ways to coproduce a healthcare service that is capable of a greater contribution to better health. We believe that the impact of COVID-19 opens an opportunity not to return to the “old normal” or develop a “new normal” based on the old, but rather to conceptually redefine what we mean by quality in healthcare, how we define each other’s roles and how we define person-centred care for individuals and communities.

## Assumptions underlying a new quality movement

Underlying our thinking has been a recognition of the benefits of understanding systems as complex adaptive phenomena, of recognising that at some level all healthcare service is coproduced by persons we sometimes call professionals and persons we sometimes call patients. They are “kin” to each other in this interdependent work ^45^.

The failure to link up the different parts of care during the pandemic, e.g. social care with healthcare, has exposed an underlying problem with the design of care. This has meant that many vulnerable people were placed at risk and potentially endured more harm. Healthcare quality and safety requires the interaction of these complex parts, continually adapting to the changing demands, each with its own complexity and each of which having to integrate at a specific time to deliver safe, good quality care. For example, the initial approach to patient safety (called Safety 1) focused on addressing adverse events and undertook linear assessments of unsafe events. These cause and effect assessments were often too simplistic to consider the complexity of causal systems at work. The progression has been to an understanding of complexity and resilience in quality and safety, with the building of resilience and constant learning, as we adapt to changing circumstances (called Safety 2). A different approach to quality is required as well ^46, 47^.

The quality and safety movement has been reactive to what has not been working and we believe that we now need to move to the concept of health and its coproduction. The concept of coproduction of quality in service systems is in its early phase of development ^48– 50^. There is a need to include people as partners and to move away from the correction of defects in disease management towards the creation of health. People, i.e. both the professionals and the patients interdependently involved, are not the problem, they are the key to a future quality model. While there has been a growing body of evidenced-based interventions, the problem has been one of implementation, spread and sustainability of interventions that have a firm evidence base ^51^. We believe that organised efforts of quality improvement and safety, be it the practice or academic research of the practice has become too technical and people cannot relate to the challenge of actually fostering better health. We need a paradigm that works in today’s real world. One that facilitates better health for individuals and communities, so that the goal of better health will be achieved. In an era where shared creation of services is key, human resources in healthcare will become one of the major challenges. Quality should include care for both persons as patients and as professionals.

## The model

**Figure 1.**
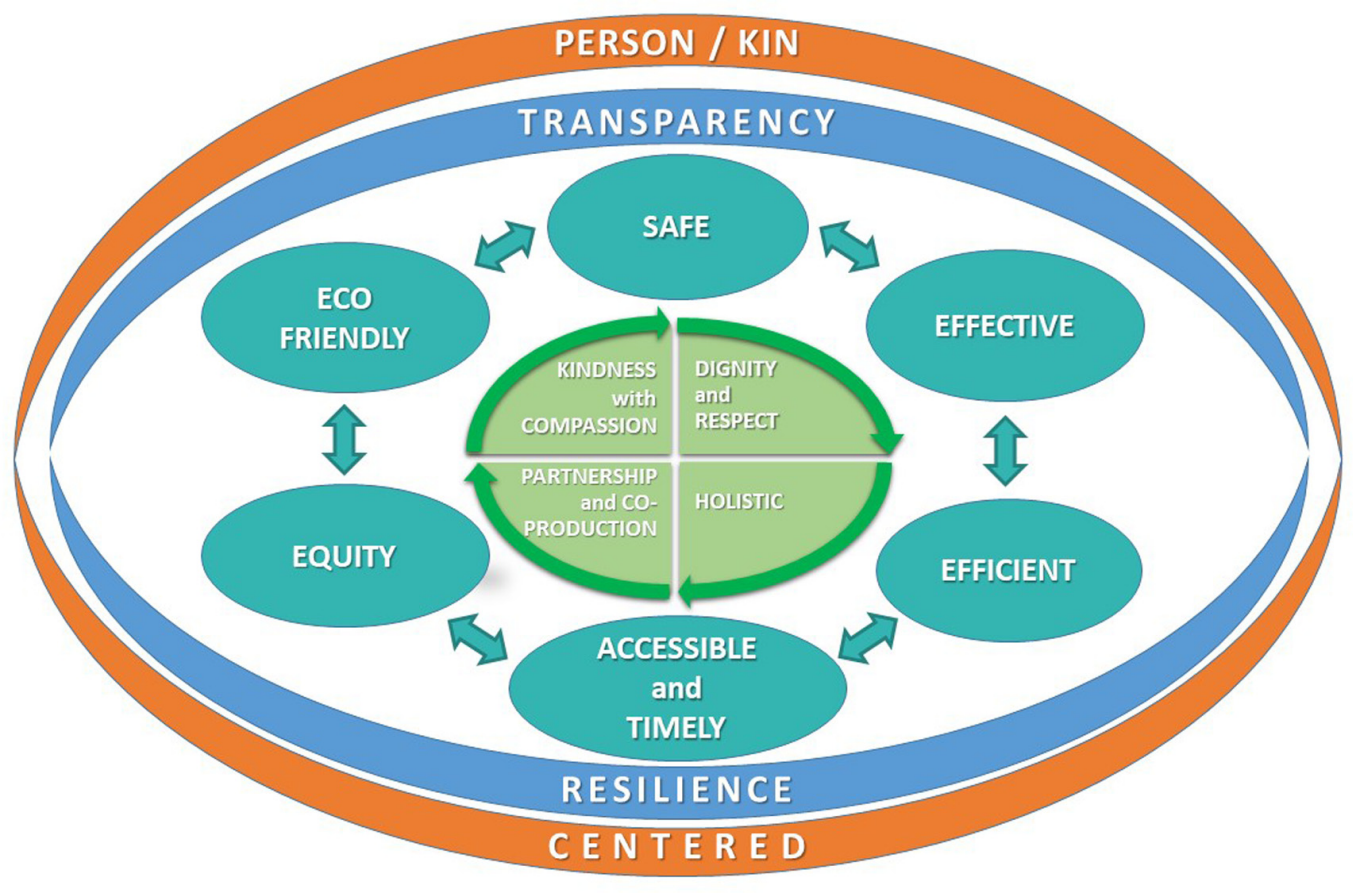
The domains of quality for the new era of health.

The six domains of quality in the IOM model no longer fit the requirements of a person-centred approach to the facilitation of health and the delivery of universal healthcare. We suggest a focus on the co-creation of better health — a quality system for the people who are working together to co-produce services that contribute to better health ( Figure 1).

The original model had person-centred care as one of the domains. We wish to further develop this by recognising the shared humanity of the people involved. The word “kin” is introduced to embody the social relationships and lived realities that surround the individuals involved, both those providing care and those receiving care. Healthcare service is not only about the person as patient or professional, but also about their family and wider social relationships. The dimension person/kin-centred surrounds every domain and is part of all that we do. The need for this approach has been demonstrated to be an essential component of the response to the pandemic. John Ballatt and colleagues suggest that “kindness [kinshipness] is ...not a ‘nice’ side issue, it is the glue of cooperation required for progress to be the most beneficial to the most people” ^45^.

We place the person at the core of quality, rather than being a separate domain. At the core are the values of healthcare, based on kindness with compassion; partnership and coproduction; dignity and respect for people and each other; where people are seen from a holistic approach, in their totality and not as a disease or an organ of the body. The central tenet is kindness, so the dimension of person-centred care is *kin-centred* as well, involving all those who are related to the person receiving and the person providing care. This approach will facilitate the coproduction of quality and safety and achievement of the other domains. This emphasis invites and expands change from “installing” technical solutions to working with people and technical solutions. Telehealth efforts make it clear that more use of digital connectivity can work and possibly become part of the extended connectivity of kin ^52, 53^. The other domains remain in place. They are transfused with person-centred care. This new way of thinking also applies to the other person involved in making the service called “healthcare.” This means that among colleagues, and certainly with regards to relationships with hierarchical supervisors, there needs to be an understanding built on kindness, dignity, respect and partnership – and it includes the holistic person.

A new domain, eco-friendly, is added to reflect the growing challenges of climate change and to introduce the need to address the challenges of sustainability, not only on organisation level, but in every contact in the micro-system ^54, 55^. We believe that being eco-friendly with a concern for climate change is central to the concept of kinship. The principle of transparency is included to surround all the technical domains, respecting the person’s right to privacy but also the right to know the data that specifically concerns themselves. Transparency is needed for providers, so that they can be open with themselves, as well as with the people to whom they deliver care. Transparency and resilience, i.e. the ability to operate with psychological safety, are the basis for the pursuit of truthful data collection, analysis and interpretation. Transparency with all our “kin” begins with professionals being transparent with each other ^56^. 

## Implication for current programmes

We believe that healthcare promotion and the delivery of healthcare must return to the core tenets of care—a form of “service”—and include the values that we have made central to the model in everything that we do. In the supplementary document we demonstrate the actions that are required to implement this new quality paradigm. Kin and person-centred care are infused in every effort to improve care, safety and effectiveness. The introduction of transparency will require a culture change in every sector of healthcare. Ecology is now a central domain, so all decisions and planning will require programmes to improve the impact on the climate and environment. Quality health services are based on what one human offers to another. These services are fundamentally a human activity, with attendant rights, responsibilities, and implications. To achieve this, we need to have high quality care for the professionals who deliver care and a redesign of systems, in order to facilitate true person and kin-centred care. In Table 2 the possible actions to be undertaken are suggested, these are not comprehensive and will be dynamic, changing in different contexts.

**Table 2.** The domains of quality and action to be taken.

Domain of quality	Patient/Kin receiving care	Person providing care	Organisation
**Person/Kin** **centred**	The care a person receives should be filled with kindness, dignity, and respect. People should be seen as a whole and their care must be coproduced. Shared decision-making and self- management are essential.	The person providing care should experience psychological safety, kindness, dignity and respect with a sense of belonging and meaning. This will facilitate the resilience or coping skills required by healthcare professionals to feel physically and mentally safe.	The core value is about quality, and kin-centred care health with meaning and purpose. Leadership is distributed to engender physical and psychological safety for all people proving care. Meaning and purpose to the work is part of all decision making and the organisation is learning from excellence and challenges.
**Safety**	Care should be free from harm, where harm is defined as *something one* *would not accept for oneself or one’s Kin* *(physical or psychological).*	Psychological safety is a central part of the culture. Proactive management of risk and learning from incidents is standard. Debriefing and support are provided after an incident.	Learning and understanding how the complexity of the system works, is a daily activity. Designing for safety using human factors is central to all operations.
**Effective**	All care follows evidence-based guidelines and standard operating procedures (SOP) where appropriate, with deviation only as per need of the person receiving care.	Reliable care is provided following SOPs to reduce unwarranted variation. Transparency on (non-)compliance to SOPs is evident.	Translating evidence-based guidelines into local protocols. Benchmarks process and outcome indicators.
**Efficient**	Unnecessary care is not provided. All care should have intended benefit.	Care provided is cost-effective, minimising duplication and waste. Clinicians constantly study processes to improve. Focus on prevention of wasteful processes. Improvement and or management methods are used to decrease waste.	Administrative waste is decreased. Constant attention to pricing and cost of care without decreasing quality is standard. Health is the outcome one aims for, rather than disease management.
**Accessible and** **Timely**	There are no delays in receiving care. Universal quality with safe access is the goal.	Working in teams to provide care. Available 24/7/365 with respect to staff wellbeing and risk of burn-out and bore-out.	Organisation of services so that they are accessible. Manage the impact of weekend- effect or out-of-office hours demand.
**Equitable**	Care is of the same quality all the time, no matter who you are and where you require care.	Seven-day week service for acute care that is fully staffed for acute care. No racism among staff. Real interprofessional care where all professionals can contribute equally.	Active programmes to decrease institutional racism, or any discrimination based on gender, ethnicity, sexuality disability etc. Focus on the Social Determinants of Health.
**Eco-friendly**	Kin and the person aim to receive care that decreases duplication, repetition and over-investigation or treatment. Decrease unnecessary consultations.	No duplication of tests. Electronic records where possible and use of digital health. Decrease disposables and consumables in all processes. Organise video-consultation to decrease need to attend clinics.	Water and energy management. Less use of plastic. Conversion to reusable energy. Active programmes for heat conservation and efficient water disposal.
**Core values**	**Patient or Kin**	**Provider**	**Organisation**
**Dignity and** **Respect**	All views are accepted and respected in all decision-making.	Practices shared decision-making. Is treated with respect by other providers from own and other disciplines. Does not see divisions of care.	Develops a culture of learning and respect. Provides a sense of belonging. Develops psychological safety of staff.
**Holistic**	Care addresses physical needs as well as spirituality and mental wellbeing in an integrated manner.	Moral compass in all activities. Treats patients as people, not as diseases and integrates care.	Breaks down the silos between levels of care so that the person experiences integrated care.
**Partnership and** **coproduction**	Be an active partner in designing health. Able to choose where and how to receive care.	Sees patients as equal partners to develop health. Coproduces health with people. Supports the involvement of patients as experts by experience.	Works across all systems in pursuit of health. Are people focused. Performs experience-based coproduction programmes.
**Kindness with** **compassion**	Appreciation of the human side of the person. Patient/Kin are kind to the provider.	Appreciation of the human side of the person. Is always kinder than necessary.	Appreciation of the human side of the person. Kindness is quality indicator in balanced scorecard.

## Conclusion

Over the past few years, there has been a growing realisation that the current design of the system of healthcare has resulted in decreased wellbeing for the professionals involved in healthcare, with increasing reports of burn-out and “bore-out” ^57^. The impact of safety events on clinicians has been documented and a meta-analysis of wellness and burn-out demonstrates the negative impact on care givers ^58, 59^. The review by the National Academies of Sciences concluded that the delivery of quality person-centred care will require a workforce whose wellbeing is paramount, which implies the dehumanisation of healthcare must be reversed ^60, 61^.

The recent focus on health inequalities and structural racism makes a change of focus more pressing with the concept of kinship reaching to the core of what it is to be a healer. This attention to relationship-as-fundamental is not new. In addition to the bridging energy for our use as we address the “schism”, we also recognise that numerous cultures across the globe have realised for centuries that this universal recognition of the importance of relationship is fundamental in all human life. Perhaps this is best known in the African philosophy of Ubuntu, where “I” am because “we” are. It is our contention that the new model of quality that we propose is the first step in this direction for policy makers, leaders and healthcare providers to explore and embrace this new way of thinking and to invite a return to a recognition of our shared humanity and the importance of kindness in healthcare for people and kin.

## Data availability

No data is associated with this article.
