# Supplementary material for: A multidimensional quality model: an opportunity for patients, their kin, healthcare providers and professionals to coproduce health
Source: F1000Res. 2021 Jul 20;9:1140. Originally published 2020 Sep 16. [Version 3] doi: 10.12688/f1000research.26368.3 (PMC8191516; doi:10.12688/f1000research.26368.3)
Supplement: Peer review history

# Peer review: Reviewer response for version 3

**DOI:** 10.5256/f1000research.58266.r89992

**Published:** 2021-09-27

**Authors:** Gro Rosvold Berntsen

**Affiliations:** 1 Norwegian center for e-health research, University hospital of North Norway, Tromsø, Norway; 2 The Primary Care Research group, Department of Community Medicine, Faculty of Health Sciences, UiT the Arctic University of Norway, Tromsø, Norway

Thank you for an excellent paper. No further comments.

Is the topic of the opinion article discussed accurately in the context of the current literature?

Partly

Are arguments sufficiently supported by evidence from the published literature?

Partly

Are all factual statements correct and adequately supported by citations?

Yes

Are the conclusions drawn balanced and justified on the basis of the presented arguments?

Yes

Reviewer Expertise:

Health service researcher with a focus on digitally supported, person-centered, integrated and proactive care.

I confirm that I have read this submission and believe that I have an appropriate level of expertise to confirm that it is of an acceptable scientific standard.

# Peer review: Reviewer response for version 2

**DOI:** 10.5256/f1000research.42565.r82799

**Published:** 2021-06-09

**Authors:** James Mountford

**Affiliations:** 1 Royal Free London NHS Foundation Trust, London, UK

This is a well-written, thought-provoking and timely addition to the literature on quality of care. The historical context around eras 1 and 2 of quality links well to the cogently set-out framework for quality which includes kindness/kin and person-centred as elements which run through the whole frame. In the UK/NHS context at least (& I expect for other health systems too), this framework will be of practical use to a range of people aiming to rebuild better in light of the pandemic. The authors should be congratulated on bringing together complex issues, multiple strands and schools of evidence into a coherent, readable and likely actionable whole.

Is the topic of the opinion article discussed accurately in the context of the current literature?

Yes

Are arguments sufficiently supported by evidence from the published literature?

Yes

Are all factual statements correct and adequately supported by citations?

Yes

Are the conclusions drawn balanced and justified on the basis of the presented arguments?

Yes

Reviewer Expertise:

Quality measurement and improvement ; System thinking ; Coproduction and kindness in health care ;

I confirm that I have read this submission and believe that I have an appropriate level of expertise to confirm that it is of an acceptable scientific standard.

# Peer review: Reviewer response for version 2

**DOI:** 10.5256/f1000research.42565.r77700

**Published:** 2021-01-28

**Authors:** Gro Rosvold Berntsen

**Affiliations:** 1 Norwegian center for e-health research, University hospital of North Norway, Tromsø, Norway; 2 The Primary Care Research group, Department of Community Medicine, Faculty of Health Sciences, UiT the Arctic University of Norway, Tromsø, Norway

I think you have responded well to my comments. I especially liked the following response which i think is key to the potential impact of this paper:

 "Firstly, person centred care is expanded to kin centred so is an overtly broader; and secondly we do not see it as a separate domain of quality by rather in every domain. We contend that other models have focussed more on the technical – e.g. patient safety is a good example of this problem in the past.”

 I think it is essential that you bring this **point out in a much clearer way in the abstract**, and possibly also in the text. This is the central change, and a much needed change in our thinking about quality of care. People-centeredness is the primary concern, which is served by our advanced biomedical knowledge and skills. We are humans first, both the patient and the provider. If our humanity is lost, the health care transaction turns into a violation of both the patient and the provider. Most of the other arguments of the paper flow from this one point and as such deserves to be highlighted.

 Good luck.

Is the topic of the opinion article discussed accurately in the context of the current literature?

Partly

Are arguments sufficiently supported by evidence from the published literature?

Partly

Are all factual statements correct and adequately supported by citations?

Yes

Are the conclusions drawn balanced and justified on the basis of the presented arguments?

Yes

Reviewer Expertise:

Health service researcher with a focus on digitally supported, person-centered, integrated and proactive care.

I confirm that I have read this submission and believe that I have an appropriate level of expertise to confirm that it is of an acceptable scientific standard, however I have significant reservations, as outlined above.

# Response

**Published:** 2021-07-19

**Authors:** Kris Vanhaecht

**Affiliations:** KU Leuven Institute for Healthcare Policy, Belgium

Thank you for the positive review. We agree with your suggestions and have added a section in the abstract and main text to emphasise the importance of the person as a person, for both the patient and the provider.

# Peer review: Reviewer response for version 1

**DOI:** 10.5256/f1000research.29109.r73483

**Published:** 2020-11-09

**Authors:** Gro Rosvold Berntsen

**Affiliations:** 1 Norwegian center for e-health research, University hospital of North Norway, Tromsø, Norway; 2 The Primary Care Research group, Department of Community Medicine, Faculty of Health Sciences, UiT the Arctic University of Norway, Tromsø, Norway

**Summary**

 The paper is an opinion piece which analyses the development of the concept of “Quality of care” over time:

- Quality (Q) 1.0 – Accreditation and Improvement cycles
- Q 2.0 – IOM. The study and practice of clinical excellence
- Q 3.0 – New model in the current paper.

An acknowledgment of several shortcomings in the current IOM dominating quality of care model inspired this new quality of care model. The current IOM model consists of six goals: Effective, efficient, patient-centered, safe, timely, and equitable care. The authors claim that the following challenges make it necessary to review the IOM model:

- The Quality Chasm is 20 years after the publication of IOM’s model for quality, not yet closed.
- The current model of care is too focused on disease and disease management and less on health.
- There is no focus on “relationships” and co-production of care in the IOM model, which is necessary for patients and professionals to “work” together towards a common good.
- The focus on general prevention is lacking.
- What added value health care provides is not explicitly addressed.

A new model for quality must attempt to correct these shortcomings. The new model builds on the IOM model but adds "Eco-friendly" as a 7th quality domain in addition to the original six IOM dimensions. It also provides a strong argument for Kinship as a core value in health care. The authors argue that Kinship is related to kindness – the sort of action you would confer upon someone of your kind. The authors underscore the importance of how both patient and professionals are persons and that although they have different roles, they have the same fundamental human needs for values that are core to the new model:

- Kindness and compassion.
- Dignity and respect
- Holistic
- Partnership- and co-production

The new model also has an outer "shell" consisting of: 

- Transparency and resilience
- Person/ Kin- Centered.

All the terms of the model are described more in detail in table 2, which shows how the authors expect each of the following “roles”: Patient-kin, Health provider, Organization, to work, or act in alignment with the goals that make up the model.

**Strengths:**

 I agree with the authors that the current IOM-model of quality of care has not brought about the expected improvements. In practice, the IOM-domains of effective and efficient, and safe care have gotten all the attention and become the primary "measures" of care quality. The softer goals of person-centered, timely, and equitable care have also received some attention, but not nearly as much as the three former.

 This paper adds some genuinely new ideas in the following areas:  

- The authors emphasize Kin-ship and how we are all first humans. It underscores even more than the many other papers on Person-centeredness in care that the foundation for success is a relationship built on trust between the person who needs care and the person who provides it. Without a robust trusting relationship, the common goal(s) of health can not be reached.
- Also, I believe that the cross-table between roles and care goals is genuinely new. It bridges the gap from the desired outcome, e.g., safety (the row-headline), the most critical resources (column headlines), and the process (Cell value). The essential resources in every care process are those mentioned in the column headings: the patient, the provider, and the Organization.
- The domain Eco-friendliness is not commonly highlighted in other publications on quality of care and adds a novel dimension to the complex concept of quality.
- The strong emphasis on the well-being of the person who provides care is not entirely new ^1^. Still, this paper gives it a more decisive role than most other similar publications on quality of care, and I believe this to be an essential contribution.

**Comments**

 The authors present their quest as a novel approach: “ **Assumptions underlying a new quality movement**." However, most of the features of Lachman's quality model, such as relationship-based care, patient-centeredness, and transparency, were highlighted in the original IOM "Quality Chasm” report (See “Simple Rules for the 21st-Century Health Care System, p. 67”).  Many other authors have also pointed out many of the same shortcomings of current care systems ^2^, ^3^, ^4^, ^5^. The terms of kindness, compassion, dignity and respect, holism, partnership, and co-production, are desired outcomes highlighted before by different authors ^6^, ^7^, ^8^, ^9^, ^10^. These authors share the concern, with Lachman *et al.*, that the current care systems are not adopting the quality chasm report's recommendations well enough, nor fast enough. It would strengthen the paper to acknowledge how this paper builds and adds to the many previous publications that have pointed this out.

 The many prior authors on this topic show that practice worldwide builds on a “too technical understanding of care quality” over a long period. The bigger question then is: Even though many express the need for qualities similar to those presented in this new model, such as person-centeredness, compassion, and empathy in current care, these authors concern does not seem to make an impact on care practices. Why are these compelling publications and analyses, many with strong arguments and support from empirical data, not making a difference? I would welcome the authors' reflection upon the following question: How will this model make a difference when other publications did not? What is it with this model that makes it stand out from previous models?

 The paper has a paragraph concerned with complexity and how linear solutions may not provide the answers we need. The illustrated version of the quality model reflects the complexity in the arrows that link every component to other components. I share this view. Furthermore, I support the authors' recommendation of co-production, where a plurality of voices participates in the change implementation.  However, change management in complex adaptive systems rely heavily on a shared vision and iterative trial-and-error implementation efforts, where measurement/ observations of "the desired change" are central ^11^. I want to challenge the authors on the consequences of making a complexity science approach to improvement: How should we identify the measurements or observations that will guide change?

 Donabedian's Structure => Process => Outcome model for understanding a care process's outcomes is still relevant. In their quality model, the authors have given us several domains that should be reflected in the care process. I would like to see the authors reflect upon the following question: If this is the process we would like to see, what changes, if any, do we need in care structures that would support such an approach?

 The authors strongly argue the need to honor the relationship between patient and provider. However, in many care processes, the only stable person is the patient. Only those conditions that are managed by the general practitioner (GP) have continuity in terms of "persons" present in the relationship. Even in the GP office, there are temps and health secretaries and nurses, so that personal continuity might not be ensured even there. For patients with more complex issues, the care process consists of a string of care providers, who, more often than not, are not fully aware of what happened last with the last provider they saw. I invite the authors to reflect upon the "patient-provider relationship" in a situation where many different persons share the role of "the provider."

 1.            Bodenheimer, T. and C.J.T.A.o.F.M. Sinsky, *From triple to quadruple aim: care of the patient requires care of the provider.* 2014. **12**(6): p. 573-576. ^1^

 2.            WHO. *Framework on integrated, people-centred health services. Report by the Secretariat*. 2016  08.08.2019]; 1-12]. Available from: Accessed at: http://apps.who.int/gb/ebwha/pdf_files/WHA69/A69_39-en.pdf?ua=1 ^2^

 3.            WHO, *The World Health Report 2000. Health Systems: Improving Performance.* 2000. ^3^

 4.            D’Avena, A., et al., *Normalizing High-Value Care: Findings of the National Quality Task Force.* NEJM Catalyst Innovations in Care Delivery, 2020. ^4^

 5.            Rich E, et al. *Coordinating Care for Adults With Complex Care Needs in the Patient-Centered Medical Home: Challenges and Solutions. White Paper *2012  08.08.2019]; Available from: Archived at: http://www.webcitation.org/73DWky5eg. ^5^

 6. *Person-centered care made simple*. 2014  08.08.2018]; Available from: Archived at: http://www.webcitation.org/73DX9aYtT ^6^

 7.            McWhinney, I.R., *Family medicine in perspective.* New England Journal of Medicine, 1975. **293**(4): p. 176-181. ^7^

 8.            Jewson, N.D., *The Disappearance of the Sick-Man from Medical Cosmology, 1770-1870.* Sociology, 1976. **10**(2): p. 225-244. ^8^

 9.            Mead, N. and P. Bower, *Patient-centredness: a conceptual framework and review of the empirical literature.* Social Science & Medicine, 2000. **51**(7): p. 1087-110. ^9^

 10.         Coulter, A., et al., *Personalised care planning for adults with chronic or long-term health conditions.* Cochrane Database of Systematic Reviews, 2015(3): p. 1-129. ^10^

 11.         Snowden, D.J. and M.E. Boone, *A leader's framework for decision making.* harvard business review, 2007. **85**(11): p. 1-9. ^11^

Is the topic of the opinion article discussed accurately in the context of the current literature?

Partly

Are arguments sufficiently supported by evidence from the published literature?

Partly

Are all factual statements correct and adequately supported by citations?

Yes

Are the conclusions drawn balanced and justified on the basis of the presented arguments?

Yes

Reviewer Expertise:

Health service researcher with a focus on digitally supported, person-centered, integrated and proactive care.

I confirm that I have read this submission and believe that I have an appropriate level of expertise to confirm that it is of an acceptable scientific standard, however I have significant reservations, as outlined above.

## References

[1] : From triple to quadruple aim: care of the patient requires care of the provider.*Ann Fam Med*.12(6) : 10.1370/afm.1713573-610.1370/afm.171325384822PMC4226781

[2] : Framework on integrated, people-centred health services.2016; Reference source

[3] : The World Health Report 2000 Health Systems: Improving Performance.2000;

[4] : Normalizing High-Value Care: Findings of the National Quality Task Force. *NEJM*.2020;

[5] Coordinating Care for Adults With Complex Care Needs in the Patient-Centered Medical Home: Challenges and Solutions. Reference source

[6] Person-centered care made simple.2014; Reference source

[7] : Family medicine in perspective.*N Engl J Med*.1975;293(4) : 10.1056/NEJM197507242930405176-8110.1056/NEJM1975072429304051134530

[8] : The disappearance of the sick-man from medical cosmology, 1770-1870.*Int J Epidemiol*.2009;38(3) : 10.1093/ije/dyp180622-3310.1093/ije/dyp18019433521

[9] : Patient-centredness: a conceptual framework and review of the empirical literature. *Social Science & Medicine*.2000;51(7) : 10.1016/S0277-9536(00)00098-81087-111010.1016/S0277-9536(00)00098-811005395

[10] : Personalised care planning for adults with chronic or long-term health conditions.*Cochrane Database Syst Rev*.2015; 10.1002/14651858.CD010523.pub2CD01052310.1002/14651858.CD010523.pub225733495PMC6486144

[11] : A leader's framework for decision making.2007;18159787

# Response

**Published:** 2021-01-04

**Authors:** Kris Vanhaecht

**Affiliations:** KU Leuven Institute for Healthcare Policy, Belgium

**Response to Gro Berntsen**

 Thank you for your valuable comments We have responded to each as follows.

1. Features such as relationship-based care, patient-centeredness, and transparency, were highlighted in the original IOM "Quality Chasm” report would strengthen the paper to acknowledge how this paper builds and adds to the many previous publications that have pointed this out      

We agree with this point . With reference to the IOM report while this has been so, the focus has been more on the product logic as we have noted and called Quality  2.0. We think that the new model addresses this issue and brings the person into the centre rather the process.

 Thank you for the references which we agree will ground the paper better within the literature.   We have added as follows:

*In this paper, we propose a new construct for defining quality of healthcare, where the aim is to meet the needs of the patient as a person, rather than meeting the needs of the healthcare system, which is as complex industry selling a product of disease management.[38] The construct builds on an often overlooked emphasis in the original IOM concept, namely that person centredness is central to quality.[1]  Some authors have focused on the need for compassion and person centredness to have a greater position in a quality framework and have noted the shortcomings of many initiatives.[39,40,41,42]  In the person centred care literature the lack of kindness and respect has been raised as  failing in our healthcare systems.[43,44,45,46,47,48] Despite the focus on the need for person centred care to be a central part of the quality system there has not been the traction required to make a difference. We believe that this is because person centred care is seen as a separate domain rather than one that is a precondition in every domain of quality.*

1. How will this model make a difference when other publications did not? What is it with this model that makes it stand out from previous models?

This is an important question that we think is difficult to answer as only time will tell. However we think there are two difference from past models. Firstly, person centred care is expanded to kin centred so is an overtly broader; and secondly we do not see it as a separate domain of quality by rather in every domain. We contend that other models have focussed more on the technical – e.g. patient safety is a good example of this problem in the past.  

1. **How should we identify the measurements or observations (of complexity) that will guide change? **

Table 3 provides many different possible interventions, each of which could be developed into a measure of the complexity and the changes required. This noted in the text

1. **If this is the process we would like to see, what changes, if any, do we need in care structures that would support such an approach? **
2. **I invite the authors to reflect upon the "patient-provider relationship" in a situation where many different persons share the role of "the provider."**

In response to the above two question, We agree there needs to be some consideration of the Donabedian model and the different relationships and have amended as follows:

*We believe that healthcare promotion and the delivery of healthcare must return to the core tenets of care—a form of “service”—and include the values that we have made central to the model in everything that we do. As one reflects on the Donabedian construct of “Structures and Process leading to Outcomes,” neither the structures nor processes we currently have designed are able to deliver a care model that could encompass the domains of quality nor kin centred approach. Healthcare will require a considerable redesign in which power is transferred to the person rather than remaining in the system. This would entail placing the people who receive care in positions of power in deciding how care should be delivered. And services be planned. As the complexity of care has redefined the way care is delivered with several providers often being involved in the delivery of care, the concept of integrating care around the person receiving care will be required with partnership and collaboration being core.*

 We have reflected on the need for leadership and have added a reference to this point. We believe that this will be ongoing work in process. As we see the model being adopted so we will learn about the complexity of implementation. Nonetheless, the outcome will be looking at health from the view of the person and kin rather from the current model based on disease.

# Response

**Published:** 2021-01-20

**Authors:** Gro Berntsen

Dear Authors,

 I think you have responded well to my comments. I especially liked the following response which i think is key to the potential impact of this paper:

 “Firstly, person centred care is expanded to kin centred so is an overtly broader; and secondly we do not see it as a separate domain of quality by rather in every domain. We contend that other models have focussed more on the technical – e.g. patient safety is a good example of this problem in the past.”

 I would expect to see this **point outlined in the abstract**, because this is the central change that you are advocating.

 Good luck.

# Peer review: Reviewer response for version 1

**DOI:** 10.5256/f1000research.29109.r71675

**Published:** 2020-10-13

**Authors:** Ross Baker

**Affiliations:** 1 Health Policy, Management and Evaluation, University of Toronto, Toronto, Canada

This article offers a substantial revision to the dominant model of healthcare quality and the measurement framework for that model, derived from the IOM Crossing the Quality Chasm report. The current model has been highly influential in the strategies and interventions developed to improve quality of care, yet frustratingly limited in practice. The authors acknowledge the ground-breaking nature of the current model (over the earlier framework that focused on standards and inspection.) They also point to a number of areas where that model has failed to yield substantive results because of poor implementation strategies that aim at technical changes rather than creating a more resilient and complex adaptive system. More profoundly, they also criticize the limitation of current themes reflected within the current model that aim at improving disease management rather than producing health, involve patients, but fail to create more effective co-production of services, and fail to create an environment that supports both providers as well as patients and caregivers.

 The arguments are complex and thought-provoking, and ambitious in scope. Still, the reader is sometimes left uncertain about the requirements of the new quality framework; more details on several important constructs would aid in clarifying these ideas. In particular, the distinction between product dominant logic and service dominant logic is tied to the ideas of co-production and kinship but the linkages between these ideas is not fully developed for those unfamiliar with the sources, and examples might help to elucidate the ways in which these constructs are intertwined. The notion of “kinship of coproducing people” deepens the understanding of how providers and patients with their caregivers might more effectively identify appropriate care, but it is unclear what this might look like in practice, and how to develop closer connections and co-production in an environment where providers are stretched to the limit by growing needs.

 A critical issue for this new framework and the individual elements within is “how do we enable the work needed to achieve quality of care as defined in this way?” The details provided in Table 2 offer detail and definitions, but little guidance of what is needed to achieve quality in these domains. Full specification is likely beyond what could be addressed in the current article, but illustrative examples might provide guidance. Since the focus of the authors is aspirational, their conclusions are more speculative than can be documented from published literature. Nonetheless, their arguments provide a useful set of ideas and provocations to refocus current efforts to improve the quality of healthcare services.

Is the topic of the opinion article discussed accurately in the context of the current literature?

Yes

Are arguments sufficiently supported by evidence from the published literature?

Partly

Are all factual statements correct and adequately supported by citations?

Yes

Are the conclusions drawn balanced and justified on the basis of the presented arguments?

Yes

Reviewer Expertise:

NA

I confirm that I have read this submission and believe that I have an appropriate level of expertise to confirm that it is of an acceptable scientific standard, however I have significant reservations, as outlined above.

# Response

**Published:** 2021-01-04

**Authors:** Kris Vanhaecht

**Affiliations:** KU Leuven Institute for Healthcare Policy, Belgium

**Response to Ross Baker**

 Thank you for the valuable review. We have identified three main issues to be addressed.

1. **Distinction between product logic and service logic **

We accept that the explanation of the distinction between product and service logic had not been as well developed as it could have been. We have added more detail to support this part of the argument . We have added a table which can provide can easier way to distinguish between n the two

 We have added as follows as well as provided a table to explain the point. Changes on page 6-7

*With the Industrial Revolution came the development of the goods/product dominant logic for manufacturing. This logic separated the producer and the consumer with progressive specialization of the producer and the production of homogenous goods with progressively more efficient methods of production. This logic became a pervasive model for the operations of organized work and was transposed into the design of healthcare where the clinician held all the knowledge and skills and provide care to the person as a  patient.*

*Today the internet connects across “separated” functions and fosters networking that obliterates the earlier separation of producer and consumer. Service-dominant logic fosters integrated resources and interactivity and collaborative work of producers and consumers for mutual value-creating work.[33]  For healthcare this implies that the distinction between clinician as the holder of knowledge no longer holds and the patient is now a person who can share in finding the solution.*

*If one considers the study of the process of production of an outcome, the logic behind the making of products, or “goods” involves linked processes. Efforts to improve those processes often uses “standardization” of the processes and their linkages. The output of the processes is usually tangible. The logic behind the making of a “service” usually involves interactive steps of professionals and beneficiary users working in dyads or networks that are needed to solve a problem, on an individual or group basis.[34,35] Therefore the service will require interaction between all parties involved*.  t.

1. **Requirements of the new framework**
2. **What the interventions may look like in practice especially where providers are stretched due to growing need**

These two points are related. A theoretical model needs to be tested and implemented. We believe the model brings together some of the current concerns regarding wellness of healthcare workers, of people  who receive care and the concepts of kindness. As the model is new it has been tested in a limited way in Belgium where it has been adopted by the local policy makers as a framework. One could also look at different quality frameworks developed  in other organization where elements of the framework are included in their definition of quality, e.g., in British Columbia. . https://bcpsqc.ca/what-is-quality/ We contend that the framework is being implemented in part and it brings together many of the current activities in to one construct. We have expanded on this  in the text and have added more on leadership as any changes will require a leadership responsive to both people receiving care and those providing care.   

*The model can be used to define and translate your own view on quality or integrate different visions and ideas into one overall framework. The multidimensional model has been piloted recently in different types of care organizations and it helped the involved clinicians and managers to define and specify the organization specific goals for the six technical domains, the umbrella domain of person and kin centred care and how to focus on the four core values. For example, to demonstrate application of the new eco-friendly domain one could preserve energy, water, resources, improve are using digital interventions and decrease carbon footprint. We invite clinical teams to use the model to examine how they can become person centred and then publish their experience so that we can coproduce the future.*
