# Supplementary material for: A multidimensional quality model: an opportunity for patients, their kin, healthcare providers and professionals to coproduce health
Source: F1000Res. 2021 Jan 19;9:1140. Originally published 2020 Sep 16. [Version 2] doi: 10.12688/f1000research.26368.2 (PMC8191516; doi:10.12688/f1000research.26368.2)
Supplement: Peer review history

# Response

**Published:** 2021-01-04

**Authors:** Kris Vanhaecht

**Affiliations:** KU Leuven Institute for Healthcare Policy, Belgium

**Response to Gro Berntsen**

 Thank you for your valuable comments We have responded to each as follows.

1. Features such as relationship-based care, patient-centeredness, and transparency, were highlighted in the original IOM "Quality Chasm” report would strengthen the paper to acknowledge how this paper builds and adds to the many previous publications that have pointed this out      

We agree with this point . With reference to the IOM report while this has been so, the focus has been more on the product logic as we have noted and called Quality  2.0. We think that the new model addresses this issue and brings the person into the centre rather the process.

 Thank you for the references which we agree will ground the paper better within the literature.   We have added as follows:

*In this paper, we propose a new construct for defining quality of healthcare, where the aim is to meet the needs of the patient as a person, rather than meeting the needs of the healthcare system, which is as complex industry selling a product of disease management.[38] The construct builds on an often overlooked emphasis in the original IOM concept, namely that person centredness is central to quality.[1]  Some authors have focused on the need for compassion and person centredness to have a greater position in a quality framework and have noted the shortcomings of many initiatives.[39,40,41,42]  In the person centred care literature the lack of kindness and respect has been raised as  failing in our healthcare systems.[43,44,45,46,47,48] Despite the focus on the need for person centred care to be a central part of the quality system there has not been the traction required to make a difference. We believe that this is because person centred care is seen as a separate domain rather than one that is a precondition in every domain of quality.*

1. How will this model make a difference when other publications did not? What is it with this model that makes it stand out from previous models?

This is an important question that we think is difficult to answer as only time will tell. However we think there are two difference from past models. Firstly, person centred care is expanded to kin centred so is an overtly broader; and secondly we do not see it as a separate domain of quality by rather in every domain. We contend that other models have focussed more on the technical – e.g. patient safety is a good example of this problem in the past.  

1. **How should we identify the measurements or observations (of complexity) that will guide change?**
** **

Table 3 provides many different possible interventions, each of which could be developed into a measure of the complexity and the changes required. This noted in the text

1. **If this is the process we would like to see, what changes, if any, do we need in care structures that would support such an approach?**
** **
2. **I invite the authors to reflect upon the "patient-provider relationship" in a situation where many different persons share the role of "the provider."**

In response to the above two question, We agree there needs to be some consideration of the Donabedian model and the different relationships and have amended as follows:

*We believe that healthcare promotion and the delivery of healthcare must return to the core tenets of care—a form of “service”—and include the values that we have made central to the model in everything that we do. As one reflects on the Donabedian construct of “Structures and Process leading to Outcomes,” neither the structures nor processes we currently have designed are able to deliver a care model that could encompass the domains of quality nor kin centred approach. Healthcare will require a considerable redesign in which power is transferred to the person rather than remaining in the system. This would entail placing the people who receive care in positions of power in deciding how care should be delivered. And services be planned. As the complexity of care has redefined the way care is delivered with several providers often being involved in the delivery of care, the concept of integrating care around the person receiving care will be required with partnership and collaboration being core.*

 We have reflected on the need for leadership and have added a reference to this point. We believe that this will be ongoing work in process. As we see the model being adopted so we will learn about the complexity of implementation. Nonetheless, the outcome will be looking at health from the view of the person and kin rather from the current model based on disease.

# Response

**Published:** 2021-01-20

**Authors:** Gro Berntsen

Dear Authors,

 I think you have responded well to my comments. I especially liked the following response which i think is key to the potential impact of this paper:

 “Firstly, person centred care is expanded to kin centred so is an overtly broader; and secondly we do not see it as a separate domain of quality by rather in every domain. We contend that other models have focussed more on the technical – e.g. patient safety is a good example of this problem in the past.”

 I would expect to see this **point outlined in the abstract** , because this is the central change that you are advocating.

 Good luck.
